# Flow Cytometry as an Alternative to Microscopy for the Differentiation of BAL Fluid Leukocytes

**DOI:** 10.1016/j.chest.2024.03.037

**Published:** 2024-03-26

**Authors:** Kai Bratke, Martin Weise, Paul Stoll, J. Christian Virchow, Marek Lommatzsch

**Affiliations:** Department of Pneumology and Critical Care Medicine, University of Rostock, Germany

**Keywords:** BAL fluid, flow cytometry, leukocytes, microscopy

## Abstract

**Background:**

Microscopy is currently the gold standard to differentiate BAL fluid (BALF) leukocytes. However, local expertise for microscopic BALF leukocyte differentiation is often unavailable in clinical practice.

**Research Question:**

Can automated flow cytometry be used instead of microscopy to differentiate BALF leukocytes?

**Study Design and Methods:**

A new automated flow cytometric method for BALF leukocyte differentiation, using four antibodies (anti-CD45, anti-CD66b, anti-HLA-DR, anti-CD52) given to human BALF in one tube, was developed and prospectively validated in 745 unselected subsequent BALF samples from patients with interstitial lung diseases (455 patients), infectious diseases (196 patients), and other diseases (94 patients). Flow cytometry and traditional microscopy were performed by separate investigators in a double-anonymized fashion. Results were compared using Spearman correlation, Deming regression, and Bland-Altman analysis.

**Results:**

There was a strong correlation between flow cytometric and microscopic results regarding macrophage/monocyte, lymphocyte, eosinophil, and neutrophil percentages in BALF (*P* < .001 for all leukocyte subpopulations). Bland-Altman analyses showed that the mean differences between the methods were ≤ 2% for all four cell types. Flow cytometric results differed less than 20% from microscopic results in more than 95% of all samples. Subgroup analyses confirmed that these results were independent from total leukocyte counts in BALF.

**Interpretation:**

We report, to our knowledge, the first validated flow cytometric method for BALF leukocyte differentiation, which can be used in clinical settings where local expertise for microscopic analysis is unavailable and which can be combined easily with lymphocyte surface marker analysis.

## Graphical Abstract

**Figure:**
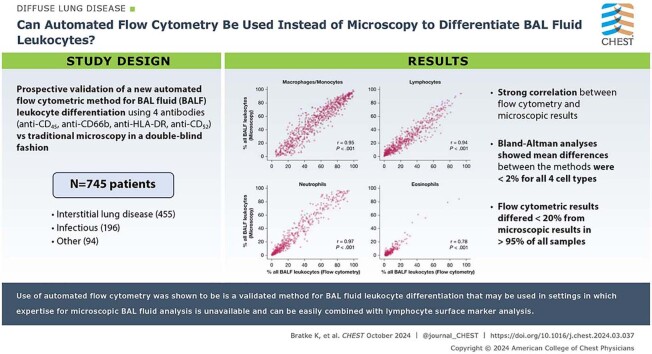

**Take-home Points**

**Study Question:** Local expertise for microscopic BAL fluid (BALF) leukocyte differentiation is often unavailable in clinical practice: Can microscopy be replaced by automated flow cytometry?

**Results:** We report, to our knowledge, the first validated automated flow cytometric method for human BALF leukocyte differentiation.

**Interpretation:** The new method bears substantial clinical relevance: it can be used in settings in which expertise for microscopic BALF analysis is unavailable, and it can easily be combined with lymphocyte surface marker analysis.

BAL, originally developed as a research tool in the 1970s,^1^, ^2^, ^3^, ^4^, ^5^ has become an essential diagnostic tool in clinical practice, primarily for the evaluation of interstitial lung diseases^6^, ^7^, ^8^ and infectious diseases,^9^^,^^10^ with acceptable safety.^11^ The exact quantification of leukocyte subpopulations in human BAL fluid (BALF), especially lymphocytes, neutrophils, and eosinophils, is an important element in the diagnostic workup in patients with interstitial lung diseases.^6^^,^^8^^,^^12^, ^13^, ^14^ Current guidelines recommend a microscopic assessment of stained cytospin preparations to quantify leukocyte subpopulations in BALF.^15^ However, microscopy of BALF cells (1) requires local expertise and experience in the microscopic analysis of BALF; (2) relies on the analysis of a small fraction of all BALF cells (typically 400-500 cells are counted^15^); and (3) cannot be combined with flow cytometric analyses of lymphocyte surface markers (eg, CD4 and CD8 in patients with suspected sarcoidosis).^16^^,^^17^

Therefore, a few research groups attempted to develop flow cytometric methods for the quantification of human BALF leukocyte subpopulations. The flow cytometric methods published by Barry et al,^18^ Hodge et al,^19^ and Shanthikumar et al^20^ were unable to measure eosinophils (or to distinguish neutrophils and eosinophils within the population of granulocytes), which limited the usefulness of these methods for clinical practice. The study by Tricas et al^21^ failed to show a clinically acceptable correlation between microscopic and flow cytometric results. The study by Pepedil-Tanrikulu et al^22^ counted only 100 cells per BALF sample in the microscopic analysis (guidelines recommend at least 400 cells^15^) and did not report the differences between the results of the microscopic and flow cytometric methods and the distribution of these differences (Bland-Altman analyses were not performed). A major limitation of the available studies is the small number of BALF samples examined (< 100 samples in all studies identified) and the selection of specific patient populations (eg, there were > 50% patients with HIV in the study by Barry et al^18^), making it difficult to predict the validity of the methods in general clinical routine. It was the aim of this study, therefore, to develop a flow cytometric method able to precisely quantify all BALF leukocyte subpopulations that are of relevance for current diagnostic algorithms (macrophages/monocytes, lymphocytes, neutrophils, and eosinophils) and to prospectively validate this method in a large group of unselected, real-world patients from routine clinical practice.

## Study Design and Methods

### BAL Fluid

In this study, we included BALF samples obtained in the Department of Pneumology at the University Clinic of Rostock (Germany) as part of the clinical routine and sent to our laboratory for quantification and differentiation of BALF leukocytes. Routine BAL was performed using flexible bronchoscopes (Olympus). The bronchoscope was wedged into a subsegment of a lobe, and portions of 20 mL sterile saline (room temperature) were instilled and the fluid of each portion was recovered by gentle aspiration. A total of 60 to 160 mL (depending on the choice of the physician) was instilled in each patient. For the development of the flow cytometric method, we used selected routine BALF samples obtained in our department between 2012 and 2013 (data not shown). For the validation study, we prospectively included unselected BALF samples sent to our laboratory over a period of 6 years. BALF samples were excluded from the analysis if (1) clinical data on the age, sex, diagnosis, or smoking status of the patients or the BALF volume were missing; (2) if only microscopy of the BALF cells was performed; or (3) if the cell concentration in the BALF sample was too low (< 0.3 × 10^4^/mL) to perform a dual leukocyte analysis (microscopy and flow cytometry). Two separate investigators performed the microscopic analysis and the flow cytometric analysis in a double-anonymized fashion. One experienced investigator analyzed all samples using the microscopic analysis, and the other investigator analyzed all samples using the flow cytometric analysis (K. B.); the respective results were not validated by another (second) investigator.

### Isolation of BALF Cells

BALF was filtered through a 70-μm nylon mesh (Greiner Bio-One, Kremsmünster, Austria). The filtered BALF was centrifuged (320 × *g*, 10 min) and then washed and resuspended with phosphate-buffered saline (PBS) (Sigma Life Science) + 2% fetal calf serum (FCS) (Pan Biotech). Subsequently, the sample was centrifuged (320 × *g*, 10 min) and then washed and resuspended with PBS + 2% FCS again. This processed cell suspension was used to differentiate leukocyte subpopulations using the microscopic and flow cytometric methods, and to count the leukocytes microscopically, using an Axio Lab.A1 microscope (Carl Zeiss).

### Microscopy

Processed BALF cells were sedimented on a microscope slide and dried on air. Cells were fixed with Hemacolor fixing solution (Merck) for 5 s, then incubated with Hemacolor color reagent red (Merck) for 3 s and finally incubated with Hemacolor color reagent blue (Merck) for 6 s. Afterward, cells were dried on air and mounted in Cytoseal XYL medium (Microm International GmbH). In every sample, a total of 500 BALF leukocytes were microscopically evaluated and classified as macrophages/monocytes, lymphocytes, neutrophils, or eosinophils (with 1000× magnification) using an Axiostar plus light microscope (Carl Zeiss). The investigator who analyzed BALF cells using microscopy was blinded for the flow cytometric results.

### Flow Cytometry: Method

Processed BALF cells were incubated in one tube with anti-CD45 fluorescein isothiocyanate (clone J33, Beckmann Coulter), anti-CD66b phycoerythrin (clone G10F5, BioLegend), anti-HLA-DR peridinin chlorophyll protein (clone L243, BD Biosciences), and anti-CD52 allophycocyanin (clone HI186, BioLegend) for 20 min. Afterward, contaminating erythrocytes were lysed with FACS lysing solution (BD Biosciences) for 10 min. Cells were centrifuged (400 × *g*, 5 min) and washed with PBS + 2% FCS. After centrifugation (400 × *g*, 5 min), cells were resuspended in PBS and analyzed with a FACS Calibur, using CellQuest Pro software (both BD Biosciences). At least 100,000 events were counted. The investigator who analyzed BALF cells using flow cytometry (K. B.) was blinded for the microscopic counts.

### Flow Cytometry: Gating Strategy

**Figure 1:**
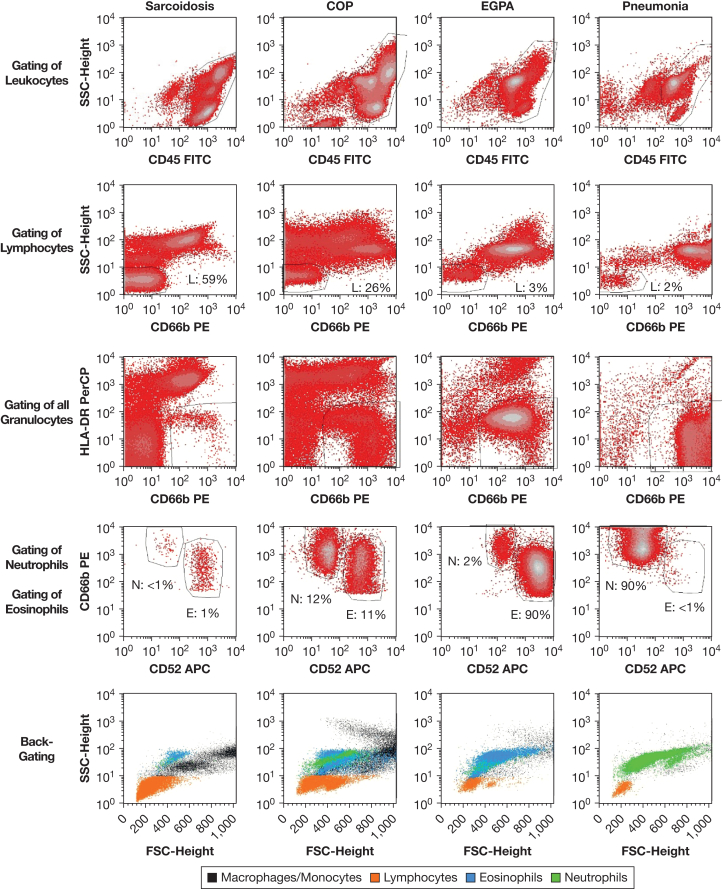
Gating of BALF cells. Density plots show the gating strategy of four representative patients with sarcoidosis (patient group 1), cryptogenic organizing pneumonia (COP) (patient group 2), eosinophilic granulomatosis with polyangiitis (EGPA) (patient group 4), or pneumonia (patient group 3). Total leukocytes are identified in a SSC/CD45-plot (first row). Then, lymphocytes are quantified by their low side scatter characteristics in a SSC/CD66b-plot (second row). All granulocytes are identified in CD66b/HLA-DR-plot by their expression of CD66b and their low or absent expression of HLA-DR (third row). In a CD66b/CD52-plot, eosinophils are identified by a high CD52 expression and neutrophils by a low CD52 expression (fourth row). The last row shows backgating: localization of lymphocytes (red), neutrophils (green), and eosinophils (blue) in the respective FSC/SSC dot plots. APC = allophycocyanin; BALF = BAL fluid; CD = cluster of differentiation; FITC = fluorescein isothiocyanate; FSC = forward scatter; SSC= side scatter; PE = phycoerythrin; PerCP = peridinin chlorophyll protein complex.

The gating strategy is shown in Figure 1. Total leukocytes were discriminated from debris or other cell types in a side scatter (SSC)/CD45-plot (first row). Lymphocytes were identified by their low side scatter characteristics and the absence of CD66b expression (second row). Total granulocytes were identified by their expression of CD66b and low to no expression of HLA-DR (third row). Afterward, eosinophils (high CD52 expression) were discriminated from neutrophils (low CD52 expression) using the difference in CD52 expression (fourth row). The percentage of macrophages/monocytes (cells from the myelomonocytic lineage) was determined by subtracting the percentages of lymphocytes, neutrophils, and eosinophils from 100%. Backgating shows distinct populations for macrophages/monocytes (black), lymphocytes (orange), neutrophils (green), and eosinophils (blue) in the forward scatter (FSC)/SSC)-plot (fifth row) (Fig 1). Figure 1 shows four examples of BALF flow cytometric analyses: BALF from patients with sarcoidosis, cryptogenic organizing pneumonia, eosinophilic granulomatosis with polyangiitis, and pneumonia. BALF samples with high neutrophil counts can contain large amounts of disrupted neutrophils: these cells are not necessarily excluded by CD45 staining and can show unspecific high staining for CD52, thus mimicking an eosinophil population (“false eosinophils”) (e-Fig 1). Therefore, it is necessary to check the correct identification of eosinophils using backgating. Backgating clearly distinguishes “false eosinophils” (low FSC signal, comparable with cellular debris) from “true eosinophils” (always a distinct population in the FSC/SSC plot with an intermediate FSC and a high SSC signal) (e-Fig 1).

## Statistical Analysis

Most statistical analyses were performed using SPSS Statistics (IBM SPSS), except for Deming regression analyses, which were performed using the R software (www.r-project.org). Most variables were not normally distributed. Therefore, the results were expressed as medians (minimum – maximum). Correlation analyses were performed using Spearman correlation coefficient. Probability values of *P* < .05 were regarded as significant. Bland-Altman plots were created to illustrate the average differences between the results of the microscopic and flow cytometric methods.^23^ The Bland-Altman method displays the difference between the results of the two measurement methods and compares the distribution of the differences. Bland-Altman plots present the difference in the percentage of BALF cells between the flow cytometric and the microscopic methods (percentage of cells determined using microscopy – the percentage of cells determined using flow cytometry) on the y-axis against the mean percentage of BALF cells determined with both methods ([% cells determined using microscopy + % cells determined using flow cytometry]/2) on the x-axis. The horizontal thick line denotes the average mean difference, and the dashed lines represent the 95% CI (lower and upper agreement limit). The 95% CI was computed using the mean difference of the results of the two methods, and the SD of this mean difference. The upper limit of the interval was calculated as follows: mean difference + (1.96 × SD of this mean difference). The lower limit of the interval was calculated as follows: mean difference – (1.96 × SD of this mean difference).

A maximum difference of 20% in the percentages of leukocyte subpopulations between the microscopic and flow cytometric method was predefined as the maximum difference that would be unlikely to decisively change clinical decision-making. For instance, it was hypothesized that, in the case of 70% neutrophils in BALF, a result of 50% neutrophils (worst case difference: –20%) or a result of 90% neutrophils (worst case difference: + 20%) would not decisively change clinical decision-making: all values would suggest BALF neutrophilia. Although in BALF samples with lower cell percentages a worst case difference of 20% would be more important (eg, 2% lymphocytes vs 22% lymphocytes), we assumed that this worst case (maximum) difference will be less likely to occur (because of lower cell percentages) and even if it did occur, it would not decisively change subsequent clinical decisions (because both values would not suggest pronounced BALF lymphocytosis).

The study was approved by the local ethics committee of Rostock (Germany) (Approval number: A 2023-0089). The research was conducted according to the principles of the World Medical Association Declaration of Helsinki.

## Results

### Patient and BALF Characteristics

A total of 745 BALF samples were included in this analysis; the characteristics of the patients and BALF samples are shown in Table 1. There was a large age range in this real-world population of patients (aged 14-90 years), nearly 40% of the patients actively smoked (Table 1). The median instilled fluid volume was 100 mL, and the median recovery 55 mL (Table 1). The whole group of patients was classified in four clinical subgroups, according to the suspected diagnosis at the time of BAL (Table 1). Group 1 (27% of the patients) were examined because of suspected hypersensitivity pneumonitis (HP) or sarcoidosis. Group 2 (34% of the patients) were examined because of other suspected interstitial lung diseases (ILDs), such as idiopathic pulmonary fibrosis, nonspecific interstitial pneumonitis, respiratory bronchiolitis ILD, or ILDs associated with systemic inflammatory conditions such as rheumatoid arthritis, systemic sclerosis, or various forms of vasculitis. Group 3 (26% of all patients) consisted of patients with acute or chronic infections, including pneumonia/lower respiratory tract infections with or without immunosuppression, TB, or infections with nontuberculous mycobacteria. Group 4 (13% of all patients) comprised patients with other diagnoses, including malignancies affecting the lung parenchyma, asthma, COPD, bronchiectasis, heart failure, or ARDS (Table 1). The percentages of macrophages/monocytes, lymphocytes, neutrophils, and eosinophils (measured with the microscopic and the flow cytometric method) in the whole group and in the four clinical subgroups are detailed in Table 2. Group 1 (HP/sarcoidosis) was characterized by increased lymphocyte percentages (median lymphocyte percentage, 26%-29%), whereas group 3 (infections) was characterized by increased neutrophil percentages (median neutrophil percentage, 25%-31%), as compared with the other groups (Table 2).

**Table 1:** Patient and General BALF Characteristics

Group	No.	Age, y	Sex, Females, %	Tobacco Use, Active, %	Instilled Volume, mL; 0.9% NaCl	Recovery, mL; BALF	Cell Counts, 10^4^ cells/mL BALF
All samples	745	64	328	294	100	55	7.3
		(14-90)	(44.0%)	(39.5%)	(60-160)	(13-120)	(0.3-312.0)
Group 1	204	55	95	71	100	55	6.5
HP/Sarcoidosis		(21-85)	(46.6%)	(34.8%)	(60-150)	(25-120)	(0.3-132.0)
Group 2	251	68	105	101	100	55	8.2
Other ILD		(27-90)	(41.8%)	(40.2%)	(60-150)	(13-100)	(0.3-125.0)
Group 3	196	64	85	86	100	53	7.9
Infections		(18-88)	(43.8%)	(44.3%)	(60-160)	(20-90)	(0.6-312.0)
Group 4	94	66	43	36	100	50	6.7
Various		(14-89)	(47.3%)	(39.6%)	(60-140)	(18-70)	(0.5-217.0)

Data are shown as medians (minimum-maximum) unless otherwise indicated. BALF = BAL fluid; HP = hypersensitivity pneumonitis; Vol. = Volume.

**Table 2:** Leukocyte Subpopulations in BALF

Group	No.	% MM	% MM	% Lym	% Lym	% Neu	% Neu	% Eos	% Eos
		MS	FC	MS	FC	MS	FC	MS	FC
All samples	745	54	52	16	16	10	9	1	1
		(0-99)	(0-100)	(0-94)	(0-94)	(0-96)	(0-94)	(0-84)	(0-90)
Group 1	204	60	57	26	29	4	4	1	1
HP/sarcoidosis		(4-97)	(0-99)	(2-88)	(0-86)	(0-94)	(0-88)	(0-34)	(0-26)
Group 2	251	54	58	14	12	11	11	2	2
Other ILD		(2-97)	(5-100)	(1-94)	(0-93)	(0-93)	(0-92)	(0-84)	(0-90)
Group 3	196	38.5	39.5	11	12	31	25	2	1
Infections		(0-95)	(0-98)	(0-94)	(0-94)	(0-96)	(0-94)	(0-19)	(0-15)
Group 4	94	61	53	12	13	8	9	1	1
Various		(2-99)	(0-99)	(1-84)	(0-81)	(0-96)	(0-92)	(0-79)	(0-76)

Percentages of macrophages/monocytes (MM), lymphocytes (Lym), Neutrophils (Neu), and Eosinophils (Eos) in BALF, as determined by microscopy (MS) or flow cytometry (FC). Data are shown as medians (minimum-maximum) unless otherwise indicated. BALF = BAL fluid; HP = hypersensitivity pneumonitis; ILD = interstitial lung disease.

### Comparison of the Results of the Microscopic and Flow Cytometric Method

**Figure 2:**
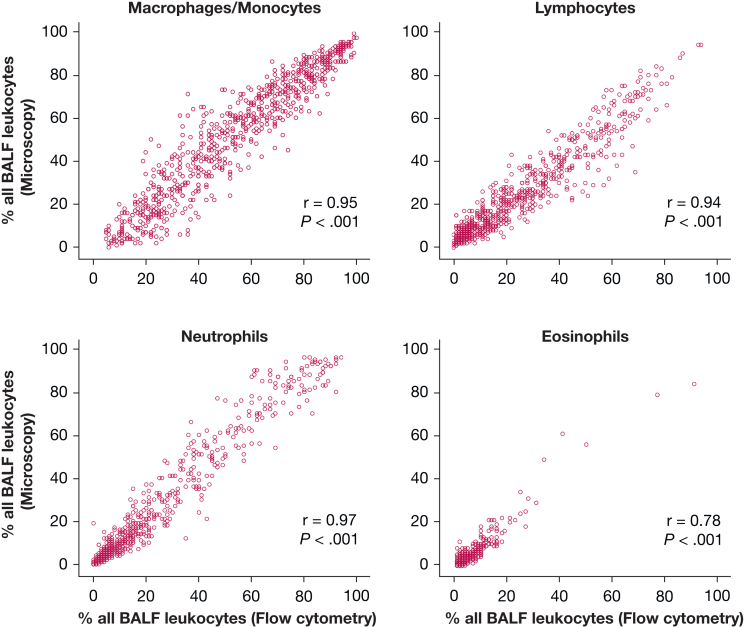
Correlations between flow cytometric and microscopic results. Shown are correlations between the percentages of specific BALF leukocyte subpopulations measured using microscopy (y-axis) and flow cytometry (x-axis). Correlation coefficients (r) were calculated using Spearman correlation analysis; the statistical significance (*P*) is shown for each correlation. BALF = BAL fluid.

**Figure 3:**
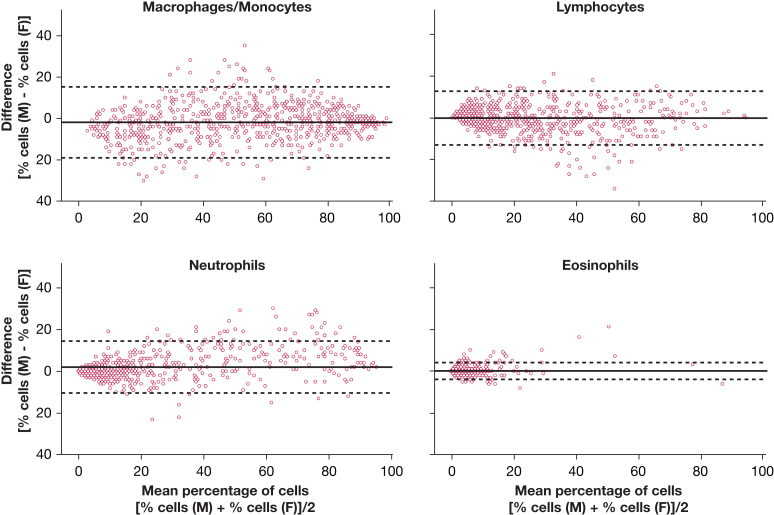
Bland-Altman analyses. Plots show the difference in the percentage of cells between the flow cytometric and the microscopic methods (% cells determined using microscopy – % cells determined using flow cytometry) on the y-axis against the mean percentage of cells determined with both methods ([% cells determined using microscopy + % cells determined using flow cytometry]/2) on the x-axis. Horizontal thick line: average mean difference, dashed lines: 95% CI (lower and upper agreement limit). F = flow cytometry; M = microscopy.

There was a strong correlation between the results of the microscopic and flow cytometric method, for macrophage/monocyte, lymphocyte, neutrophil, or eosinophil percentages (*P* < .001 for all correlations) (Fig 2). To analyze the mean differences of the two measurement methods, Bland-Altman analyses were then performed. These Bland-Altman analyses showed that the mean differences between the methods were ≤ 2% for all BALF leukocyte subpopulations (macrophages/monocytes, –1.9%; lymphocytes, –0.2%; neutrophils, 2.0%; eosinophils, 0.1%) (Fig 3). The 95% CI of the differences between the methods (lower and upper agreement limit) was within the predefined 20% margin in all BALF leukocyte subpopulations: macrophages/monocytes (lower limit, –19%; upper limit, +15%), lymphocytes (lower limit, –13%; upper limit, +13%), neutrophils (lower limit, –10%; upper limit, +14%), eosinophils (lower limit, –4%; upper limit, +4%) (Fig 3). We then analyzed whether the agreement between the two methods was influenced by total leukocyte counts (the concentration of leukocytes) in BALF. For this analysis, the whole group was divided into two subgroups: a group with leukocyte counts lower than the median value of the whole group (< 7.3 × 10^4^ cells/mL BALF: “low BALF leukocyte count group,” n = 372 BALF samples) and a group with leukocyte counts above the median BALF cell count (≥ 7.3 × 10^4^ cells/mL BALF: “high BALF leukocyte count group,” n = 373 BALF samples) (e-Figs 2, 3). In the high BALF leukocyte count group, the 95% CIs of the differences between the methods (lower and upper agreement limit) were as follows: macrophages/monocytes (lower limit, –20%; upper limit, +11%), lymphocytes (lower limit, –12%; upper limit, +13%), neutrophils (lower limit, –10%; upper limit, +16%), eosinophils (lower limit, –3%; upper limit, +4%) (e-Fig 2). In the low BALF leukocyte count group, the 95% CIs of the differences between the methods (lower and upper agreement limit) were as follows: macrophages/monocytes (lower limit, –16%; upper limit, +18%); lymphocytes (lower limit, –14%; upper limit, +11%); neutrophils (lower limit, –10%; upper limit, +12%); eosinophils (lower limit, –4%; upper limit, +4%) (e-Fig 3). Deming regression analyses revealed correlation coefficients exceeding 0.9 for all cell types, again indicating a strong agreement between the methods (e-Fig 4). Although intercepts for all cell types, except for eosinophils, deviated significantly from zero, these deviations were not clinically relevant. All intercepts were below 5% and often below 1%, remaining well within the predefined range of 20% (e-Fig 5).

## Discussion

This investigation is the first to report a validated flow cytometric method for automated leukocyte differentiation in human BALF. It was validated in a prospective, double-anonymized study, using a large number of unselected BALF samples with very different cellular components. In this broad spectrum of real-world samples, flow cytometric results correlated closely with the results of standard microscopy (mean differences between the results of flow cytometry and microscopy, ≤ 2% for all leukocyte subpopulations). In addition, more than 95% of all flow cytometric results differed less than 20% from the microscopic results (predefined margin), and this finding was independent from total leukocyte concentrations in BALF. Thus, we provide a validated and robust flow cytometric tool for the assessment of BALF leukocyte subpopulations in daily clinical practice.

Microscopy is currently the gold standard for the measurement of BALF leukocyte subpopulations in humans.^15^ However, there is a need to develop an alternative flow cytometric method for leukocyte differentiation in human BALF. Local expertise for microscopic BALF analyses is frequently not available in clinical practice, whereas laboratory facilities using flow cytometry in daily routine (eg, for differential blood analyses) are regularly available. In addition, the flow cytometry can assess large numbers of leukocytes (≥ 100,000 cells) in a short time, whereas microscopy covers only a small fraction (≤ 500 cells) of all BALF cells, which cannot exclude selection bias. Furthermore, the microscopic (visual) classification of each cell is subjective (dependent on the judgment and experience of the individual specialist), whereas the flow cytometric analysis is based on a standardized gating procedure. Finally, flow cytometric analysis of BALF leukocytes can easily be combined with flow cytometric analyses of lymphocyte surface markers, which is used for specific indications (eg, analysis of the expression of the T cell markers CD4 and CD8 in patients with suspected sarcoidosis.^16^^,^^17^ Over the last 20 years, there have been a few attempts to establish flow cytometric methods for human BALF leukocyte differentiation.^18^, ^19^, ^20^, ^21^, ^22^ However, all previous investigations examined only small numbers of selected BALF samples.^18^, ^19^, ^20^, ^21^, ^22^ In addition, the studies either failed to measure specific cell populations (especially eosinophils)^18^, ^19^, ^20^ or had methodologic problems,^21^^,^^22^ hampering a transfer of the methods into clinical practice.

Therefore, we present a new, practical method in which a hitherto unpublished combination of four antibodies is given to BALF cells in one tube: anti-CD45 (discriminating leukocytes from debris and other cell populations), anti-CD66b (also known as carcinoembryonic antigen-related cell adhesion molecule 8, a granulocyte marker,^24^ discriminating granulocytes from lymphocytes), anti-HLA-DR (discriminating granulocytes, which are HLA-DR negative, from HLA-DR positive leukocytes), and CD52 (discriminating eosinophils, which strongly express CD52, from neutrophils that are characterized by a low CD52 expression^25^). Using our four-step gating approach, we were able to precisely quantify all BALF leukocyte subpopulations essential for clinical practice (macrophages/monocytes, lymphocytes, neutrophils, eosinophils). Moreover, the results of this method proved to be comparable to the results of standard microscopy in 745 unselected, real-world BALF samples with a large variety of cell concentrations and cell distributions. Thus, we postulate that our new flow cytometric method is a robust and valid alternative to microscopy in clinical practice. Regarding expenses, overall costs for both methods might be similar. Although supplies might be more costly for flow cytometry (because of the costs for the specific antibodies), personnel costs might be higher for microscopy, especially in high-income countries (because of costs for the employment of a dedicated and well-trained specialist for BALF microscopy).

There are several limitations of our flow cytometric method as compared with traditional microscopy. First, the results of the two investigators (one for microscopy and one for flow cytometry) were not validated by another (second) investigator. Second, microscopic evaluation of the BALF cells holds the opportunity to detect other potential cell populations in BALF, such as tumor cells or bacteria, which are not detected by our flow cytometric method. However, BALF from patients with suspected tumors is usually also sent to a pathologist, and BALF from patients with suspected infections is usually also sent to a microbiology laboratory. In addition, rare leukocytes in BALF, such as dendritic cells or basophils, which are not measured by the current method, can be detected using other flow cytometric methods.^26^^,^^27^ Third, specific morphological changes of leukocyte subpopulations (eg, macrophages), which can be described using microscopy, are not identified by our flow cytometric method. However, as compared with the central role of lymphocyte, neutrophil, or eosinophil counts in BALF for diagnostic algorithms, morphological changes of these leukocytes are less vital for routine clinical decisions. Thus, our flow cytometric method does provide the key information required in clinical routine: the exact percentage of lymphocytes, neutrophils, and eosinophils among BALF leukocytes.^15^ Some outliers (beyond the predefined maximum difference of 20%) were found when comparing the results of the two methods. However, these outliers represented only a very small minority (less than 2% of all BALF samples), and there was no consistent pattern of cell counts or cell percentages attributable to this small group of outliers. Therefore, we postulate that the results of the new flow cytometric method are indeed comparable to the results of traditional microscopy in most BALF samples.

## Interpretation

We report a robust flow cytometric method for automated BALF leukocyte differentiation as an alternative to microscopy. This method can be combined with lymphocyte surface marker analysis and can be used in settings in which expertise for microscopic BALF analysis is unavailable.

## Funding/Support

This study was funded by the University of Rostock (Germany).

## Financial/Nonfinancial Disclosures

None declared.

## Supplementary Data

e-Online Data

e-Online Data

e-Online Data

e-Online Data

e-Online Data

e-Online Data
