# Supplementary figures and images for: Flow Cytometry as an Alternative to Microscopy for the Differentiation of BAL Fluid Leukocytes
Source: Chest. 2024 Mar 26;166(4):793–801. doi: 10.1016/j.chest.2024.03.037 (PMC11492222; doi:10.1016/j.chest.2024.03.037)

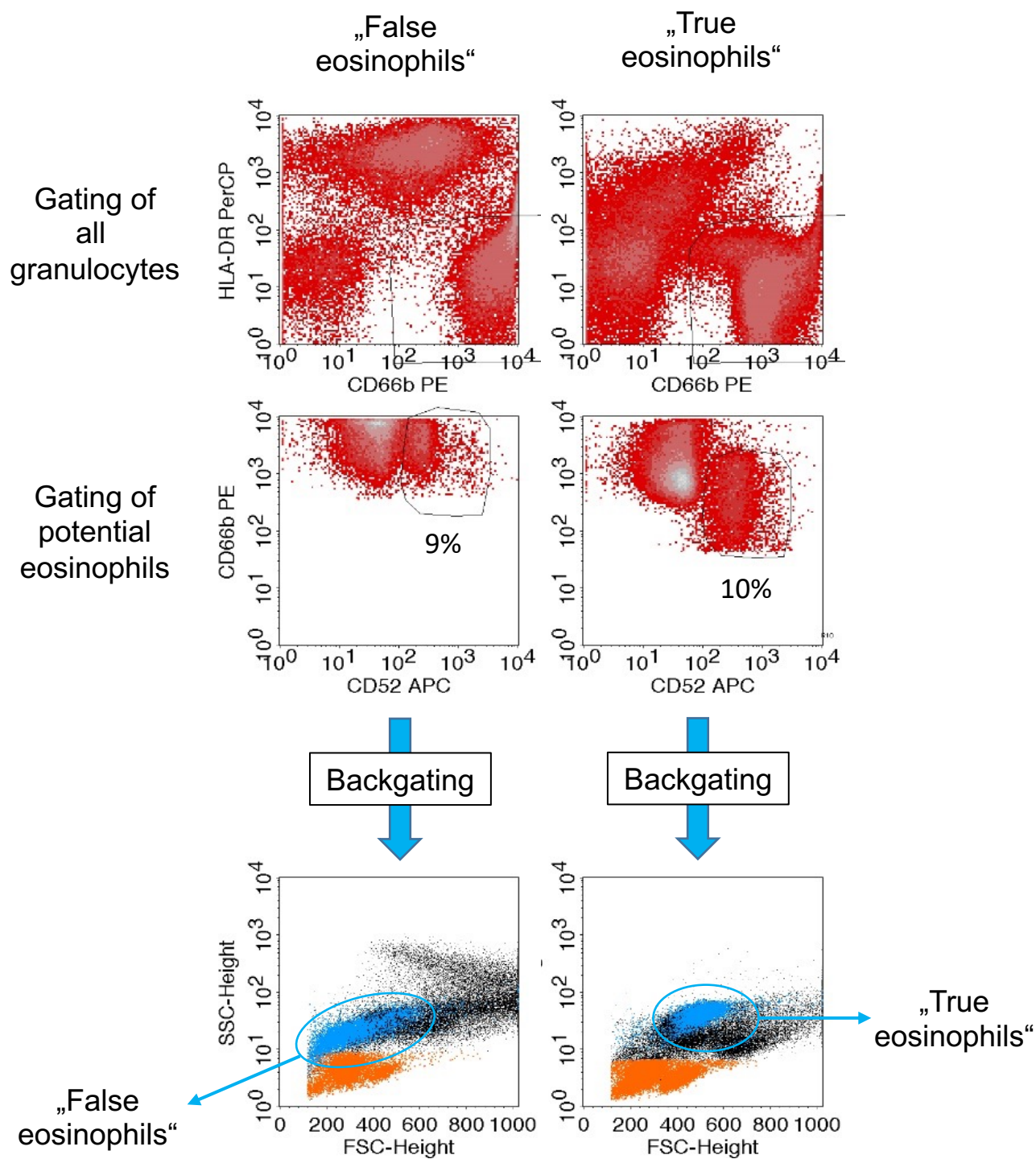

Supplement: e-Online Data [file mmc1.pdf]

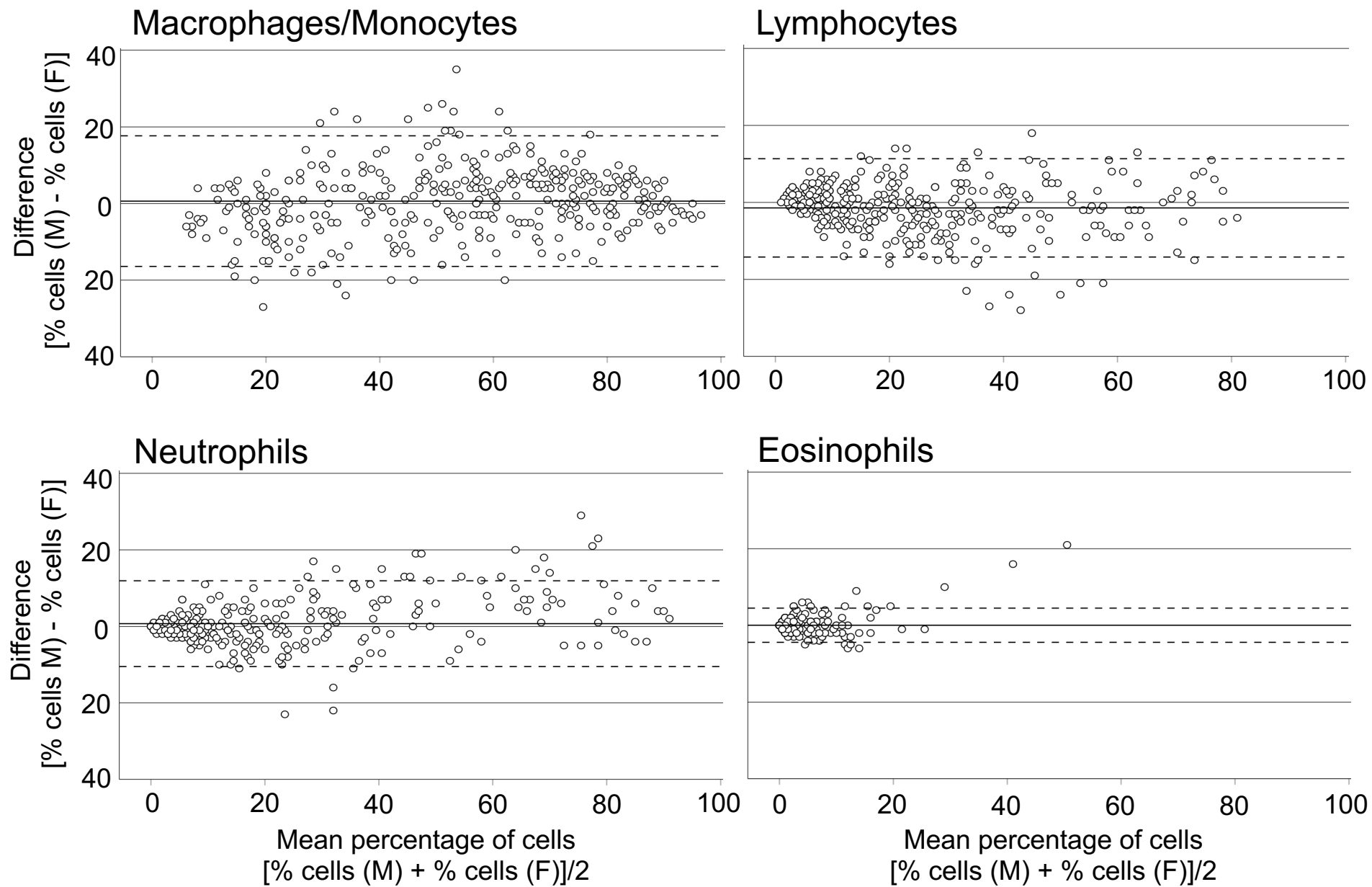

Low BALF leukocyte count group

Supplement: e-Online Data [file mmc3.pdf]

## Macrophages/Monocytes

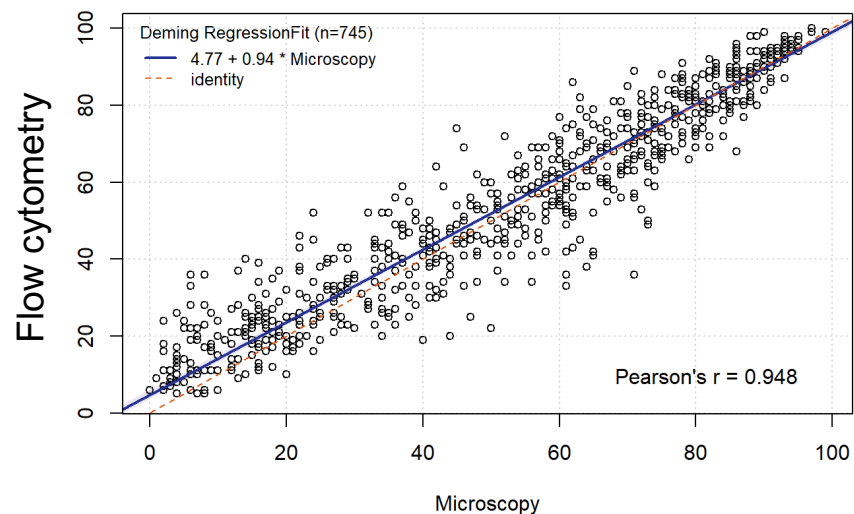

## Lymphocytes

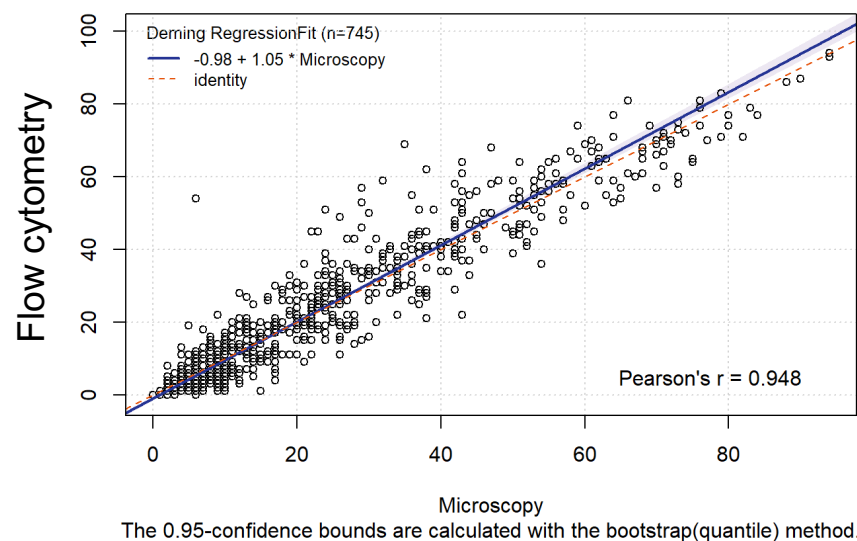

## Neutrophils

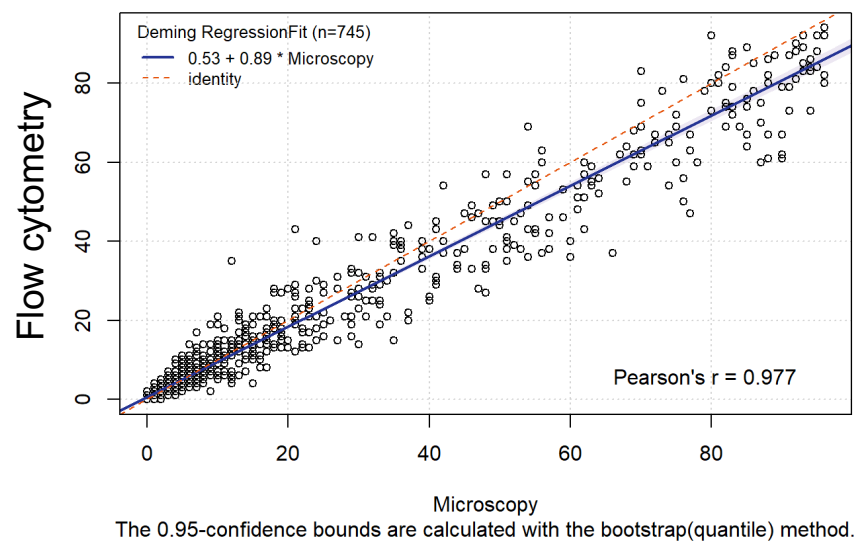

## Eosinophils

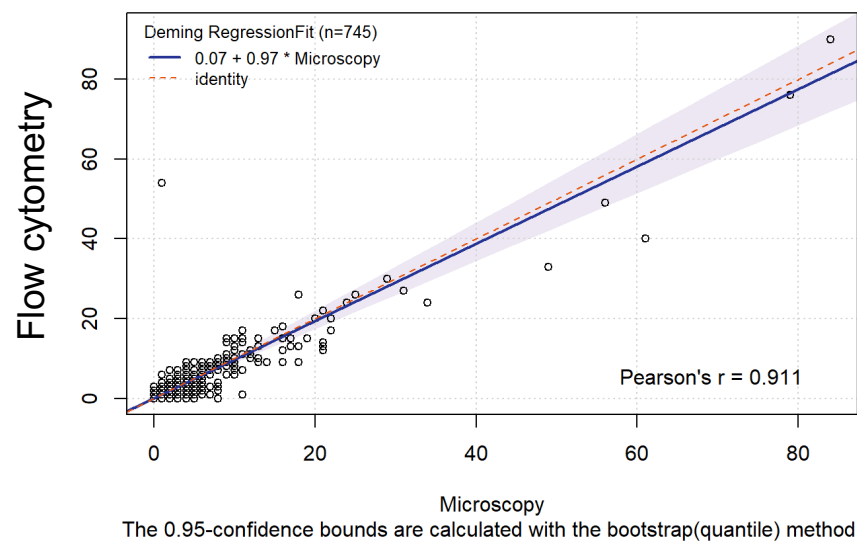

Supplement: e-Online Data [file mmc4.pdf]
