# Supplementary material for: Flow Cytometry as an Alternative to Microscopy for the Differentiation of BAL Fluid Leukocytes
Source: Chest. 2024 Mar 26;166(4):793–801. doi: 10.1016/j.chest.2024.03.037 (PMC11492222; doi:10.1016/j.chest.2024.03.037)
Supplement: e-Online Data [file mmc2.pdf]

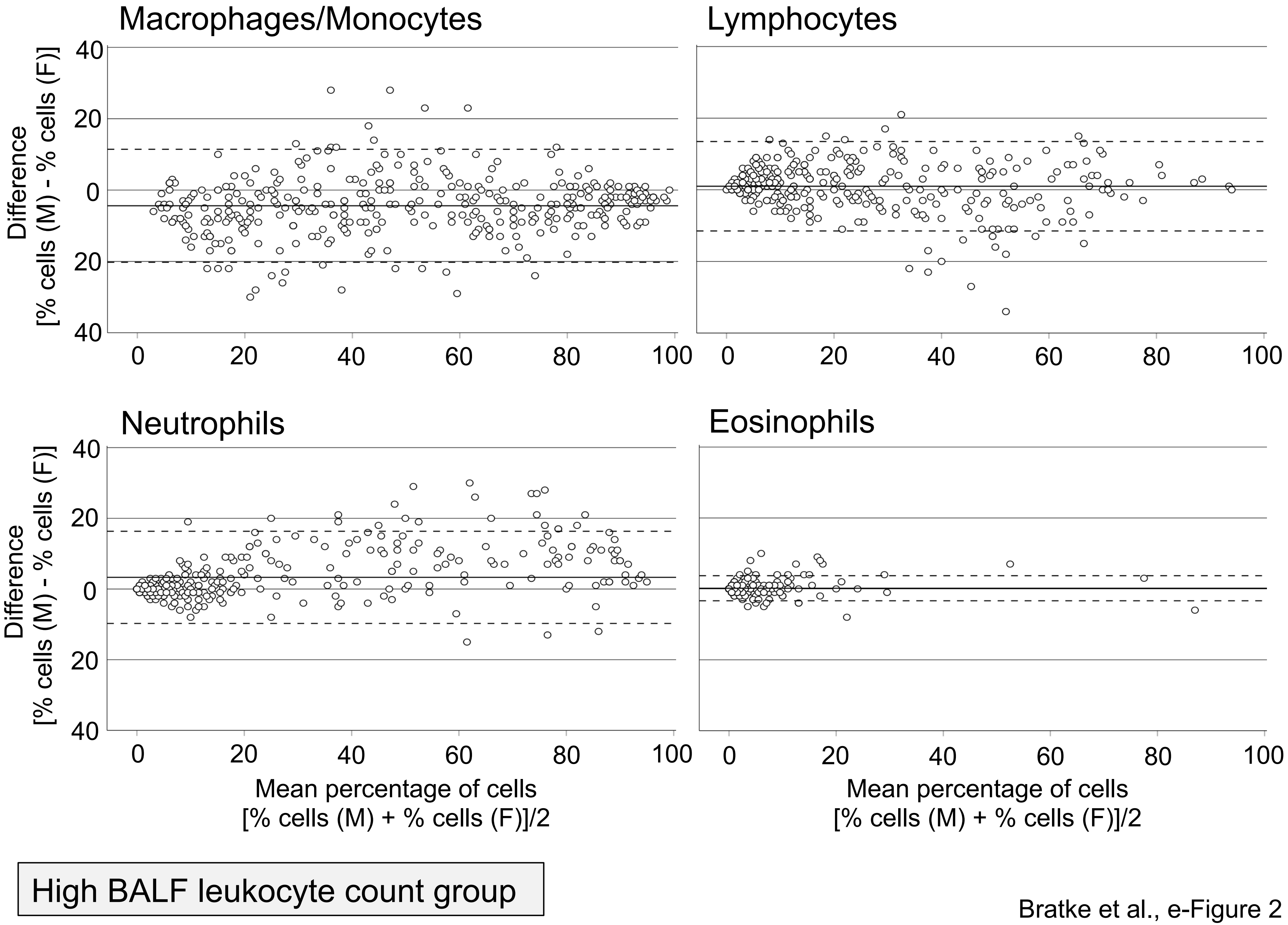

Bratke et al., e-Figure 2
