# Supplementary material for: Flow Cytometry as an Alternative to Microscopy for the Differentiation of BAL Fluid Leukocytes
Source: Chest. 2024 Mar 26;166(4):793–801. doi: 10.1016/j.chest.2024.03.037 (PMC11492222; doi:10.1016/j.chest.2024.03.037)
Supplement: e-Online Data [file mmc5.pdf]

Deming regression analyses: Intercepts and slopes

BALF leukocyte subpopulation	Coefficient	Estimate [95% confidence interval]
**Macrophages/ Monocytes**	Intercept	4.77 [3.69; 6.06]
	Slope	0.94 [0.92; 0.96]
**Neutrophils**	Intercept	0.53 [0.19; 0.89]
	Slope	0.89 [0.87; 0.91]
**Eosinophils**	Intercept	0.07 [-0.23; 0.36]
	Slope	0.97 [0.85; 1.11]
**Lymphocytes**	Intercept	-0.98 [-1.51; -0.43]
	Slope	1.05 [1.02; 1.09]

Bratke et al., e-Figure 5
