# Supplementary material for: Flow Cytometry as an Alternative to Microscopy for the Differentiation of BAL Fluid Leukocytes
Source: Chest. 2024 Mar 26;166(4):793–801. doi: 10.1016/j.chest.2024.03.037 (PMC11492222; doi:10.1016/j.chest.2024.03.037)
Supplement: e-Online Data [file mmc6.doc]

**Flow cytometry as an alternative to microscopy for the differentiation**

**of bronchoalveolar lavage fluid leukocytes**

Kai Bratke, Martin Weise, Paul Stoll, J. Christian Virchow and Marek Lommatzsch

**ONLINE ONLY MATERIAL**

**e-Figure 1: Gating of true and false eosinophils**

BALF samples with high neutrophil counts can contain large amounts of disrupted neutrophils: these cells are not necessarily excluded by CD45 staining and can show unspecific high staining for CD52, thus mimicking an eosinophil population (“false eosinophils”). Therefore, it is necessary to check the correct identification of eosinophils using backgating. Total granulocytes are identified by their expression of CD66b and low to no expression of HLA-DR (first row). Afterwards, potential eosinophils (high CD52 expression) are discriminated from neutrophils (low CD52 expression) using the difference in CD52 expression (second row). Backgating clearly distinguishes “false eosinophils” (low FSC signal, comparable with cellular debris) from “true eosinophils” (always a distinct population in the FSC/SSC plot with an intermediate FSC and a high SSC signal).

**e-Figures 2 and 3: Bland-Altmann analyses stratified by BALF leukocyte counts**

Bland-Altman-Plots show the difference in the percentage of cells between the flow cytometric and the microscopic methods (% cells determined using microscopy - % cells determined using flow cytometry) on the y-axis against the mean percentage of cells determined with both methods ([% cells determined using microscopy + % cells determined using flow cytometry]/2) on the x-axis. Horizontal thick line: average mean difference, dashed lines: 95% confidence interval (lower and upper agreement limit). Shown are Bland-Altman-Plots of the subgroup with leukocyte counts above the median BALF cell count (≥ 7.3 x 10^4^ cells /ml BALF: “high BALF leukocyte count group”, n = 373 BALF samples, e-Fig. 2) and of the subgroup with leukocyte counts lower than the median value of the whole group (< 7.3 x 10^4^ cells /ml BALF: “low BALF leukocyte count group”, n = 372 BALF samples, e-Fig. 3).

**e-Figures 4 and 5: Deming regression analyses**

Deming regression analyses were performed for all BALF leukocyte subpopulations (n = 745 BALF samples), scatterplots are shown for every cell type, correlating the results of flow cytometry (y-axis) and microscopy (x-axis), with regression lines and coefficient estimates (e-Fig. 4). The intercepts and slopes are detailed for every BALF leukocyte subpopulation (e-Fig. 5).
